# Extension of efficacy range for targeted malaria-elimination interventions due to spillover effects

**DOI:** 10.1038/s41591-024-03134-z

**Published:** 2024-07-04

**Authors:** Jade Benjamin-Chung, Haodong Li, Anna Nguyen, Gabriella Barratt Heitmann, Adam Bennett, Henry Ntuku, Lisa M. Prach, Munyaradzi Tambo, Lindsey Wu, Chris Drakeley, Roly Gosling, Davis Mumbengegwi, Immo Kleinschmidt, Jennifer L. Smith, Alan Hubbard, Mark van der Laan, Michelle S. Hsiang

**Affiliations:** 1 Department of Epidemiology and Population Health, Stanford University, Stanford, CA, USA; 2 Chan Zuckerberg Biohub, San Francisco, CA, USA; 3 Division of Biostatistics, University of California, Berkeley, Berkeley, CA, USA; 4 Malaria Elimination Initiative, Global Health Group, University of California, San Francisco, San Francisco, CA, USA; 5 PATH, Seattle, WA, USA; 6 Multidisciplinary Research Centre, University of Namibia, Windhoek, Namibia; 7 Department of Infection Biology, London School of Hygiene and Tropical Medicine, London, UK; 8 Department of Disease Control, London School of Hygiene and Tropical Medicine, London, UK; 9 University of Namibia, Windhoek, Namibia; 10 MRC International Statistics and Epidemiology Group, Department of Infectious Disease Epidemiology, London School of Hygiene and Tropical Medicine, London, UK; 11 Wits Research Institute for Malaria, Wits/SAMRC Collaborating Centre for Multi-Disciplinary Research on Malaria, School of Pathology, Faculty of Health Sciences, University of the Witwatersrand, Johannesburg, South Africa; 12 Southern African Development Community Malaria Elimination Eight Secretariat, Windhoek, Namibia; 13 Department of Epidemiology and Biostatistics, University of California, San Francisco, San Francisco, CA, USA

**Keywords:** Malaria, Developing world, Epidemiology

## Abstract

Malaria-elimination interventions aim to extinguish hotspots and prevent transmission to nearby areas. Here, we re-analyzed a cluster-randomized trial of reactive, focal interventions (chemoprevention using artemether–lumefantrine and/or indoor residual spraying with pirimiphos-methyl) delivered within 500 m of confirmed malaria index cases in Namibia to measure direct effects (among intervention recipients within 500 m) and spillover effects (among non-intervention recipients within 3 km) on incidence, prevalence and seroprevalence. There was no or weak evidence of direct effects, but the sample size of intervention recipients was small, limiting statistical power. There was the strongest evidence of spillover effects of combined chemoprevention and indoor residual spraying. Among non-recipients within 1 km of index cases, the combined intervention reduced malaria incidence by 43% (95% confidence interval, 20–59%). In analyses among non-recipients within 3 km of interventions, the combined intervention reduced infection prevalence by 79% (6–95%) and seroprevalence, which captures recent infections and has higher statistical power, by 34% (20–45%). Accounting for spillover effects increased the cost-effectiveness of the combined intervention by 42%. Targeting hotspots with combined chemoprevention and vector-control interventions can indirectly benefit non-recipients up to 3 km away.

## Abstract (web summary)

In a re-analysis of a cluster-randomized trial on malaria-control measures, the combined intervention reduces infections up to 3 km from the point of intervention, increasing efficacy and cost-effectiveness of the intervention.

## Main

In the past decade, there has been renewed attention towards global malaria eradication, and many countries have set targets for the elimination of local malaria transmission^1^. In southern Africa, eight countries hope to achieve malaria elimination by 2030 as part of the Elimination Eight Initiative. Yet global progress has plateaued: the annual number of global malaria cases has increased from 231,000 in 2015 to 249,000 in 2022, and the number of malaria deaths per 100,000 has remained nearly the same (15.0 in 2015, 14.3 in 2022)^2^.

The ideal malaria-elimination intervention would prevent not only disease among recipients, but also onward transmission to nearby non-recipients through spillover effects (that is, herd effects or indirect effects^3,4^), which some vaccines can do^3,5–11^. Earlier studies have reported spillover effects for mass drug administration for trachoma^12,13^, school-based deworming^14^, insecticide-treated bed nets^15–18^ and indoor residual spraying for malaria^19^. In the 1990s, studies estimated spillover effects from community-wide distributions of insecticide-treated bed nets^15–18^; these effects are likely to differ at present, because net coverage, insecticide resistance and mosquito behavior patterns have changed. A more recent study of spillover effects of indoor residual spraying was not randomized and might be subject to residual confounding^19^. Furthermore, to our knowledge, no studies have estimated spillover effects of malaria interventions designed for low-transmission settings approaching elimination.

When an intervention reduces disease among intervention non-recipients, accounting for spillover effects can substantially increase cost-effectiveness^20,21^. Identifying cost-effective interventions is crucial to elimination and eradication efforts because these endeavors are projected to be substantially more expensive than existing malaria-control programs in the medium term^22^. Even after elimination, countries must maintain intensive surveillance and promptly respond to imported cases to prevent re-establishment.

In settings approaching malaria elimination, the World Health Organization recommends interventions that are ‘reactive’—delivered soon after a confirmed malaria case is detected—and ‘focal’—delivered to higher risk individuals who reside near the case^23^. A recent cluster-randomized trial in Namibia found that reactive, focal chemoprevention and vector control substantially reduced malaria incidence and prevalence^24^. Spillover effects of these interventions are plausible: chemoprevention might reduce transmission to nearby areas by preventing secondary cases near index cases, and vector control can reduce the mosquito population near malaria cases. To shed light on whether focal interventions reduce transmission to nearby uninfected or asymptomatic individuals who did not receive interventions, we re-analyzed this cluster-randomized trial to separately estimate direct effects among intervention recipients and spillover effects among nearby non-recipients. Our approach can be used to estimate the spillover effects of other interventions, such as malaria vaccines.

## Results

### Study design

**Fig. 1:**
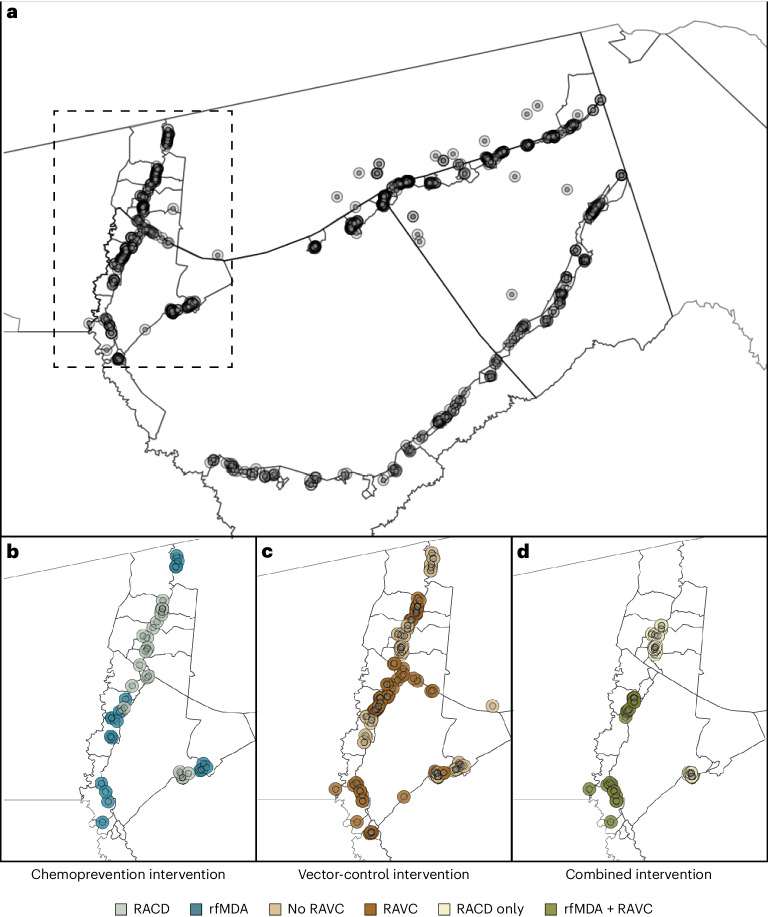
Map of target areas and spillover zones in the study site. **a**, All index cases during the study period. Black lines indicate borders of enumeration areas. The centroid of each circle is the residence location of a treated index case. Inner circles indicate 500-m target areas where interventions were delivered. Outer circles indicate the 1-km radius around each index case in which spillover effects were estimated in the primary analyses. Index-case locations were clustered along major roads, where the majority of households were located in the study site. The dashed line encloses the region shown in **b**–**d**, showing index cases during the follow-up periods with the largest number of treated index cases. **b**–**d**, Examples of analytic cohorts from a single follow-up period (that is, subsample of person-time) in a subset of the study area for each comparison of study arms. **b**, Index cases in the RACD and rfMDA arms are shown (5-week period, 25 April 2017–30 May 2017). **c**, Index cases in the no-RAVC and RAVC arms are shown (6-month period, 1 January 2017–30 June 2017). **d**, Index cases in the RAVC and rfMDA and RAVC arms are shown (6-month period, 1 January 2017–30 June 2017).

We analyzed data from a previously reported cluster-randomized trial of focal malaria interventions conducted in the Zambezi region of Namibia from 1 January 2017 to 31 December 2017 (NCT02610400)^24^ (Fig. 1). The region has low *Plasmodium falciparum* (*Pf*) malaria transmission^25^. The trial used a two-by-two factorial design in which 56 clusters were randomized to four arms in a 1:1:1:1 allocation ratio: (1) reactive case detection (RACD), (2) reactive focal mass drug administration (rfMDA) only, (3) reactive vector control (RAVC) and RACD, and (4) RAVC and rfMDA. rfMDA included presumptive treatment with artemether–lumefantrine to individuals in target areas (Supplementary Table 1). RACD included testing with rapid diagnostic tests and treatment with artemether–lumefantrine and single-dose primaquine for those who tested positive. RAVC included indoor residual spraying (IRS) with pirimiphos-methyl. The trial delivered interventions in ‘target areas’ within approximately 500 m of confirmed malaria cases detected through passive surveillance.

Primary analyses estimated effects on malaria incidence. We restructured the data into analytic cohorts to mimic data that would have been generated in a ring trial, in which index cases are randomized to interventions as they arise. This design is ideal for measuring spillover effects of a reactive, focal intervention because it allows for spatial and temporal buffers to be included around each intervention^26^. By contrast, cluster-level analyses of cluster-randomized trials could fail to capture fine-scale direct effects and spillover effects because they include all individuals, some of whom could be too far from interventions to plausibly experience spillover effects. Further, cluster-level analyses do not account for heterogeneity in the number of interventions delivered per cluster, which is common in low-transmission settings with a high degree of spatial and temporal clustering of infections.

**Extended Data Fig. 1:**
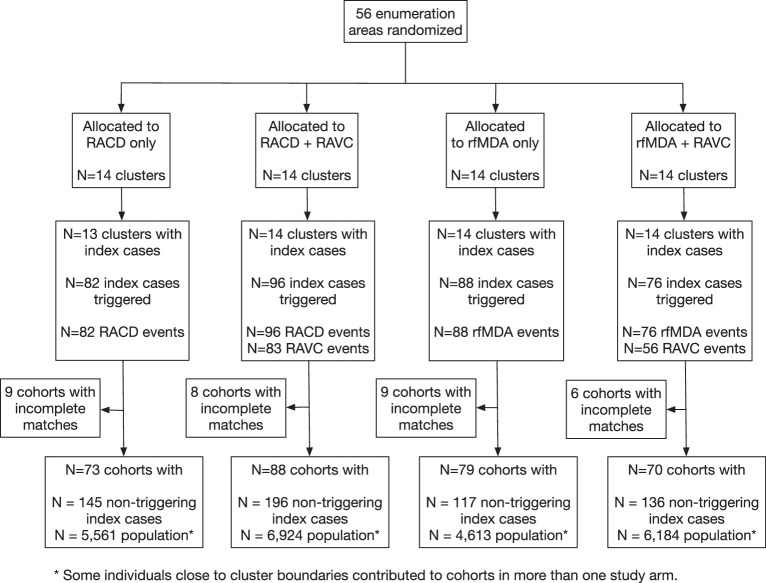
Diagram of study randomization, index cases, and population by arm. RACD: reactive case detection. rfMDA: reactive, focal mass drug administration. RAVC: reactive vector control.

Each analytic cohort included individuals residing within 1 km of each index case, to capture the area and time period in which we expected each intervention to reduce infections in intervention recipients (direct effect) and secondary transmission to nearby non-recipients (spillover effect) (Fig. 1, Extended Data Fig. 1 and Methods). For comparisons of rfMDA and RACD interventions, analytic cohorts designed to measure direct effects included 35 person-days, starting on the date of intervention delivery, to capture the length of the intrinsic incubation period for *Pf* malaria^27^; analytic cohorts for spillover effects also included 35 person-days, but started 21 days from the date of intervention delivery to capture secondary cases. For RAVC interventions, analytic cohorts for direct effects included 6 person-months because IRS can remain effective for an entire transmission season^28^; analytic cohorts for spillover effects included the period from 17 days to 6 months after intervention delivery (Methods).

We also estimated spillover effects on malaria prevalence. The analysis used cluster-level data measured using quantitative PCR (qPCR) in a cross-sectional survey at the end of the malaria season in the original trial (May to August 2017). In contrast to incidence analyses, which captured any effects within the period immediately after intervention, prevalence analyses captured effects of cumulative interventions near the end of the trial, as well as symptomatic and asymptomatic malaria cases that did not necessarily present at health clinics.

### Enrollment and baseline characteristics

The trial assessed 102 enumeration areas for eligibility as study clusters, and 56 met inclusion criteria. The analysis excluded one cluster in the RACD arm that did not have any index cases and thus did not receive any interventions (Extended Data Fig. 1). In 2016, intervention clusters had higher malaria incidence than did control clusters, and a similar pattern was observed among analytic cohorts. When comparing clusters with analytic cohorts, the mean population per study cluster was larger than that of the analytic cohorts, and baseline malaria incidence was higher in the cohorts than in the clusters (Supplementary Table 2). We had expected that this would be the case because analytic cohorts included only the areas of study clusters within 1 km around index cases. Other baseline characteristics were similar between study clusters and analytic cohorts. Among intervention recipients and non-recipients, malaria incidence in 2016 was higher in the arms including rfMDA and RAVC than in the RACD and no-RAVC arms; other baseline characteristics were balanced across study arms when comparing intervention recipients and non-recipients (Supplementary Table 3).

### Intervention delivery

During the study period, there were 1,118 eligible index cases, of which 984 resulted in 342 intervention events owing to clustering of index cases in space and time. Interventions could not be delivered within 5 weeks of presentation for 134 of the eligible index cases because of a heavy case load. The mean number of interventions delivered per cluster was 6 (range, 1–17). The proportion of eligible individuals receiving interventions was 89% for RACD, 87% for rfMDA and 89% for RAVC.

### Effects on malaria incidence

**Fig. 2:**
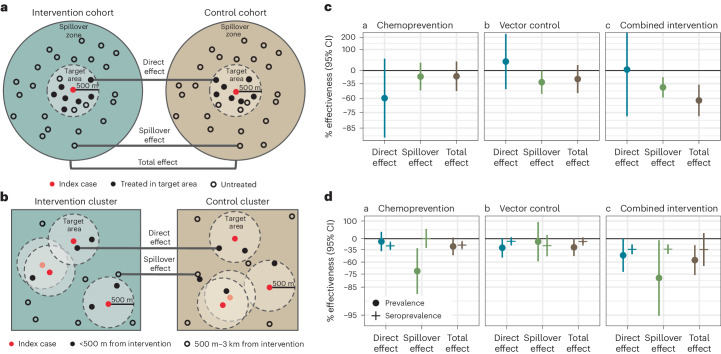
Effects of reactive, focal malaria interventions. **a**, Definition of effects in incidence analyses. **b**, Definition of effects in prevalence analyses. **c**, Effects on incidence estimated with hierarchical TMLE. Direct-effects analysis includes recipients treated in target zones, spillover-effects analysis includes intervention non-recipients up to 1 km from an index case and total-effects analysis includes all individuals (intervention recipients and non-recipients) up to 1 km from an index case. The chemoprevention analyses compared rfMDA versus RACD; the vector-control analyses compared RAVC versus no RAVC; the combined-intervention analyses compared rfMDA and RAVC versus RACD only. All incidence outcome models were fit with cohort-level data, except for models of spillover effects of the combined intervention (chemoprevention and vector control *n* = 310 cohorts; combined intervention *n* = 143 cohorts, 38,048 individuals for spillover effects of the combined intervention). Models were adjusted for covariates that were screened separately for each model using a likelihood ratio test. The chemoprevention analysis included the period of 0–35 days following index-case detection for direct effects and 21–56 days for spillover effects. The vector control and combined-intervention analyses included the period of 0–6 months following index-case detection for direct effects and 17 days–6 months for spillover effects. Total-effects analyses include the person-time for the analyses of direct effects and spillover effects. The upper bound of the 95% CI for the combined-intervention direct effect was truncated from its original value of 381%. **d**, Effects on prevalence estimated with TMLE using individual-level data; standard errors were adjusted for clustering at the enumeration-area level. See *n* values for prevalence analyses in Supplementary Table 8, and for seroprevalence analyses in Supplementary Table 10. Models were unadjusted because there were fewer than 30 observations within the strata of the intervention and outcome. Direct-effects analysis includes individuals who resided within 500 m of any intervention recipients; spillover-effects analysis includes individuals with no intervention recipients within 500 m and any intervention recipients within 500 m–3 km; and total-effects analysis include individuals with any intervention recipients <3 km during the study. In **c** and **d**, data are presented as percentage effectiveness = (1 – RR) × 100%, in which RR is the incidence ratio or prevalence ratio between study arms. Vertical bars indicate 95% CIs, not accounting for potential outcome correlation.

We estimated three types of effects on the cumulative incidence of locally acquired, confirmed *Pf* malaria: (1) direct effects among intervention recipients in target areas within 500 m of confirmed malaria index cases, (2) spillover effects among non-recipients within 1 km of index cases, and (3) total effects among all individuals within 1 km of index cases (Fig. 2a). We measured effects of (1) the chemoprevention intervention by comparing arms with rfMDA versus RACD, (2) the vector-control intervention by comparing arms with RAVC versus those without RAVC, and (3) the combined intervention by comparing the rfMDA and RAVC versus the RACD-only arms.

To estimate direct effects and spillover effects, we used hierarchical targeted maximum likelihood estimation (TMLE), a doubly-robust, semi-parametric method that adjusts for potential confounders using ensemble machine learning^29^. This approach is appropriate for cluster-level interventions in which outcomes in the same cluster could be statistically dependent on one another^30–32^ (Methods). We adjusted for covariates, such as baseline malaria incidence and population size, to account for differences in baseline characteristics between study arms (Supplementary Table 3).

We did not find evidence of direct effects among intervention recipients within 500 m of index cases for any intervention comparison. Analyses of direct effects were restricted to intervention recipients within 500 m of index cases, resulting in limited sample size and statistical power with confidence intervals including the null, which indicates that direct effects may exist but were not detected in this study (Fig. 2c and Supplementary Table 4).

We found evidence of spillover effects among intervention non-recipients up to 1 km away from interventions for the combined chemoprevention and vector-control interventions (incidence reduction, 43%; 95% CI, 21–58%) (Fig. 2c and Supplementary Table 4). For the chemoprevention intervention, we did not find evidence of a spillover effect, and for the vector-control intervention, the confidence spillover-effect estimate included the null (incidence reduction, 32%; 95% CI, 0–65%).

**Extended Data Fig. 2:**
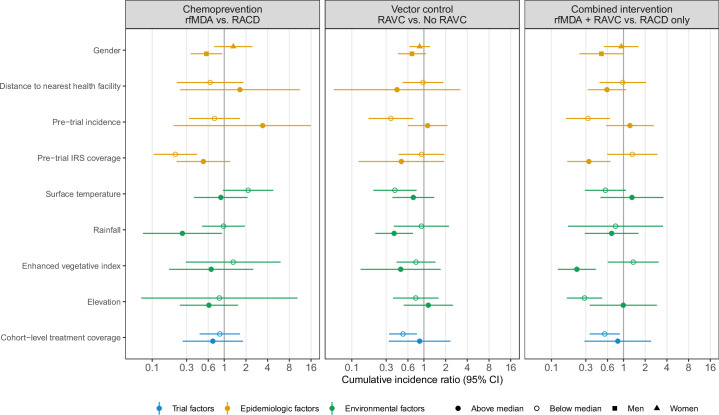
Spillover effect estimates on cumulative incidence within subgroups. Data are presented as cumulative incidence ratios between study arms with horizontal bars to indicate 95% confidence intervals not accounting for potential outcome correlation. The chemoprevention analyses compared rfMDA vs. RACD; the vector control analyses compared RAVC vs. no RAVC; the combined intervention analyses compared rfMDA + RAVC vs. RACD only. Cumulative incidence ratios were estimated with hierarchical TMLE. All outcome models were fit with cohort-level data except for models of spillover effects of the combined intervention. Models were adjusted for covariates that were screened separately for each model using a likelihood ratio test. Models for the combined intervention were unadjusted because there were fewer than 30 observations within strata of the intervention and outcome. The chemoprevention analysis includes the period from 0–35 days following index case detection for direct effects and 21–56 days for spillover effects. The vector control analysis includes the period from 0–6 months following index case detection for direct effects and 17 days to 6 months for spillover effects. Spillover effect includes intervention non-recipients up to 1 km from an index case. Analyses of the chemoprevention and vector control interventions included N = 72,830 individuals and N = 310 cohorts; analyses of the combined intervention included N = 38,048 individuals and N = 143 cohorts. For the chemoprevention intervention, confidence interval upper bounds were truncated at 16 for pre-trial incidence above the median (observed value: 46) and elevation below the median (observed value: 23).

We evaluated spillover-effect heterogeneity by cluster-level malaria incidence and IRS coverage before the trial, surface temperature, rainfall, the enhanced vegetative index, elevation, cohort-level treatment coverage and gender. Across interventions, spillover effects were consistently more protective when the pre-trial annual malaria incidence was below the median (<14 cases per 1,000 people) (Extended Data Fig. 2 and Supplementary Table 5). For example, the combined intervention reduced incidence by 68% (95% CI, 35–84%) when baseline incidence was below the median, but there was no effect when baseline incidence was above the median. This suggests that these focal interventions could be more effective in lower-transmission settings, which tend to have more focal malaria transmission. Intervention spillover effects were stronger for the chemoprevention and vector-control interventions when median monthly rainfall exceeded 24 mm, which might reflect environmental conditions that favor mosquito breeding^33^ (Extended Data Fig. 2). Spillover effects of the chemoprevention intervention were present for men but not women.

**Extended Data Fig. 3:**
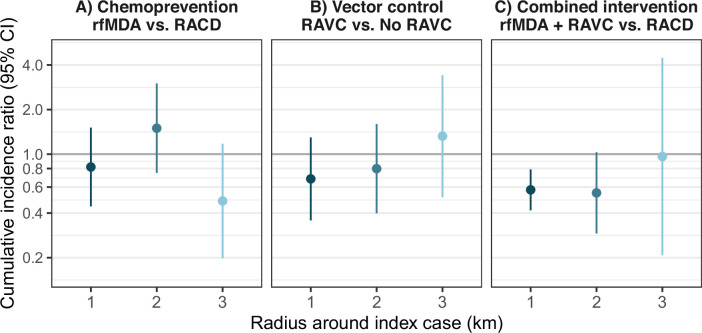
Sensitivity analyses for spillover effects on cumulative incidence of malaria with different distance radii. **a**) Chemoprevention (rfMDA vs. RACD). **b**) Vector control (RAVC vs. no RAVC). **c**) Combined intervention (rfMDA + RAVC vs. RACD only). Data are presented as cumulative incidence ratios between study arms with vertical bars to indicate 95% confidence intervals not accounting for potential outcome correlation. For rfMDA and RACD arms, the primary analysis includes the period from 0–35 days following index case detection for direct effects and 21–56 days for spillover effects (1 km N individuals = 81,082, N cohorts = 310; 2 km N individuals = 59,166, N cohorts = 294; 3 km N individuals = 46,224, N cohorts = 293). For rfMDA+RAVC and RAVC only arms, the primary analysis includes the period from 0–6 months following index case detection for direct effects and 17 days to 6 months for spillover effects (1 km N individuals = 41,962, N cohorts = 143; 2 km N individuals = 24,973, N cohorts =136; 3 km N individuals = 20,249, N cohorts = 138). Total effects analyses include the person-time for the direct effects and spillover effects analyses. Direct effect includes intervention recipients in target zone. Spillover effect includes intervention non-recipients up to 1 km from an index case in the primary analysis and up to 2 km or 3 km in sensitivity analyses. Total effect includes all individuals (intervention recipients and non-recipients) up to 1 km from the index case in the primary analysis and up to 2 km or 3 km in sensitivity analyses. Includes cohort-level analyses for all estimates except spillover effects of the combined intervention. All outcome models were fit with cohort-level data except for models of spillover effects of rfMDA vs. RACD and rfMDA + RAVC vs. RACD only.

**Extended Data Fig. 4:**
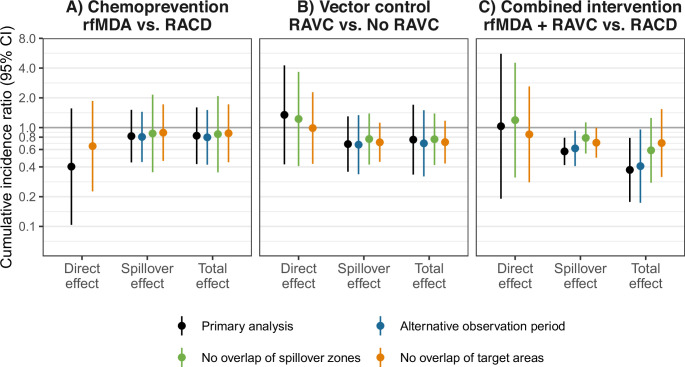
Sensitivity analyses for effects on cumulative incidence of malaria. **a**) Chemoprevention (rfMDA vs. RACD). **b**) Vector control (RAVC vs. no RAVC). **c**) Combined intervention (rfMDA + RAVC vs. RACD only). Data are presented as cumulative incidence ratios between study arms with vertical bars to indicate 95% confidence intervals not accounting for potential outcome correlation. For rfMDA and RACD arms, the primary analysis includes the period from 0–35 days following index case detection for direct effects and 21–56 days for spillover effects; the alternative observation period analysis includes the period from 0–21 days following index case detection for direct effects and 21 to 42 days for spillover effects. For rfMDA + RAVC and RAVC only arms, the primary analysis includes the period from 0–6 months following index case detection for direct effects and 17 days to 6 months for spillover effects; the alternative observation period analysis includes the period from 0–7 days following index case detection for direct effects and 17 to 90 days for spillover effects. Total effects analyses include the person-time for the direct effects and spillover effects analyses. Direct effect includes intervention recipients in target zone. Spillover effect includes intervention non-recipients up to 1 km from an index case. Total effect includes all individuals (intervention recipients and non-recipients) up to 1 km from index case. Sensitivity analyses for no overlap of spillover zones excluded any cohorts whose spillover zones overlapped spatially or temporally with other spillover zones. Sensitivity analyses for no overlap of target areas excluded any cohorts whose target areas overlapped spatially or temporally with other target areas. Some direct effects models could not be fit due to data sparsity. All outcome models were fit with cohort-level data except for models of spillover effects of rfMDA vs. RACD and rfMDA + RAVC vs. RACD only. Ns reported in Supplementary Table 7.

**Extended Data Fig. 5:**
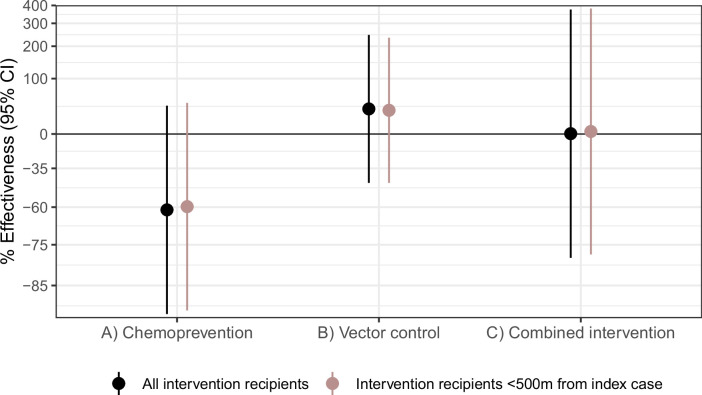
Sensitivity analyses for direct effects including all intervention recipients. Data are presented as % effectiveness = (1-RR) x 100%, where RR is the incidence ratio between study arms with vertical bars to indicate 95% confidence intervals not accounting for potential outcome correlation. The chemoprevention analyses compared rfMDA vs. RACD; the vector control analyses compared RAVC vs. no RAVC; the combined intervention analyses compared rfMDA + RAVC vs. RACD only. The observation period was 0–35 days for the chemoprevention analysis and 0–6 months for vector control and combined intervention analyses. Black points indicate estimates from analyses including all intervention recipients, regardless of whether they resided within the target zone within 500 m of index cases. Mauve points indicate estimates from analyses restricting to intervention recipients within 500 m of index cases that triggered interventions. Analyses were performed at the cohort level. For analyses of chemoprevention and vector control interventions, N = 8,351 individuals, N = 310 cohorts; for analyses of the combined intervention, N = 3,961 individuals, N = 143 cohorts.

We performed several sensitivity analyses for the primary analysis, which examined incidence within 1 km of index cases. When we conducted spillover-effect analyses using 2- and 3-km radii around index cases to account for mosquito dispersal over longer distances^34,35^, we did not find evidence of spillover effects (Extended Data Fig. 3). This could have resulted from increased overlap between study cohorts when using larger radii or lower infection intensity farther from index cases that triggered interventions. When using a shorter follow-up period in which intervention effects might have been stronger (Methods), spillover-effect estimates were similar (Extended Data Fig. 4 and Supplementary Table 6). When we repeated direct-effects analyses including the <3% of intervention recipients who resided >500 m from index cases, the results were nearly the same (Extended Data Fig. 5).

Overlap among analytic cohorts could have resulted in statistical dependence between outcomes (Fig. 1 and Supplementary Table 7). When using alternative standard errors to account for this dependence (Methods), the confidence intervals widened, but there was still evidence of spillover effects and total effects for the combined interventions (Supplementary Table 4). When we excluded overlapping cohorts from the analysis, results were similar for the chemoprevention and vector-control interventions and were attenuated towards the null for the combined intervention (Extended Data Fig. 4). This is likely because, in areas with overlapping cohorts, there were more malaria cases and a higher proportion of the population received interventions.

### Effects on malaria prevalence

Analyses of direct effects on prevalence included individuals who resided within 500 m of any intervention recipients; spillover effects included individuals who did not live within 500 m of an intervention recipient but did live within 500 m to 3 km of least one recipient; total effects included individuals with at least one intervention recipient within 3 km (Fig. 2b). We estimated prevalence ratios using TMLE and adjusted standard errors for clustering at the enumeration-area level.

**Fig. 3:**
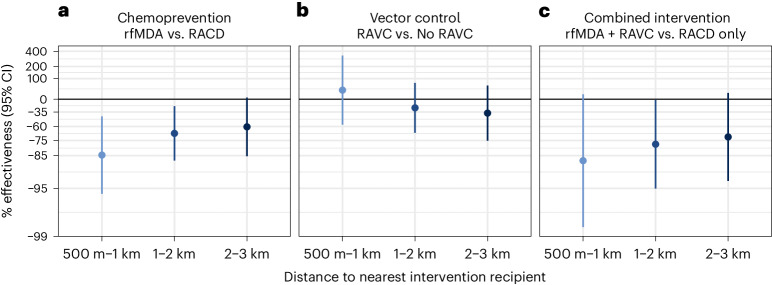
Spillover effects of reactive, focal malaria interventions on prevalence by distance to nearest intervention recipient. Spillover-effects analysis includes individuals with no intervention recipients within 500 m and any intervention recipients within the different distance radii. Data are presented as percentage effectiveness = (1 – RR) × 100%, where RR is the prevalence ratio between study arms. Vertical bars indicate 95% confidence intervals. **a**, Effects of the chemoprevention intervention (rfMDA versus RACD; *n* individuals 1 km, 245; 2 km, 435; 3 km, 367). **b**, Effects of the vector-control intervention (RAVC versus no RAVC; *n* individuals 1 km, 245; 2 km, 435; 3 km = 367). **c**, Effects of the combined intervention (rfMDA and RAVC versus RACD only; *n* individuals 1 km, 98; 2 km, 261; 3 km, 242). Effects on prevalence were estimated with TMLE using individual-level data; standard errors were adjusted for clustering at the enumeration-area level. Models were adjusted for covariates that were screened separately for each model using a likelihood ratio test. Models were unadjusted within strata of the intervention and outcome because there were fewer than 30 observations.

We found weak evidence of a direct effect for the combined intervention, but the confidence interval included the null (prevalence ratio, 0.52; 95% CI, 0.27–1.00) (Fig. 2d and Supplementary Table 8). There was no evidence of direct effects for the separate chemoprevention and vector-control interventions. There was evidence of spillover effects: among non-recipients near intervention recipients, the chemoprevention intervention reduced prevalence by 72% (95% CI, 31–88%), and the combined intervention reduced it by 79% (95% CI, 6–95%). To assess whether spillover effects varied geographically, we conducted a subgroup analysis that was stratified by distance to the nearest intervention. For the chemoprevention intervention, spillover effects were stronger closer to interventions (prevalence reduction 500 m to 1 km from interventions, 85%; 95% CI, 44–96%; prevalence reduction 1–2 km from interventions, 68%; 95% CI, 21–87%). For the combined intervention, point estimates also decreased as distance to the nearest intervention increased, but confidence intervals included the null (Fig. 3). For the chemoprevention and combined interventions, there was also evidence of spillover effects on the prevalence of households with multiple malaria cases (Supplementary Table 9).

### Effects on malaria seroprevalence

We also investigated whether there were effects on seroprevalence of early transcribed membrane protein 5 antigen (Etramp5.Ag1), an indicator of recent malaria infection^36^ that was measured by Luminex in the cross-sectional survey. There was evidence of direct effects on seroprevalence for the chemoprevention (seroprevalence reduction, 25%; 95% CI, 14–34%) and combined interventions (seroprevalence reduction, 34%; 95% CI, 10–42%) (Fig. 2d and Supplementary Table 10). There was a spillover effect among intervention non-recipients for the combined intervention on seroprevalence by (seroprevalence reduction, 34%; 95% CI, 20–45%).

### Cost-effectiveness

To inform policy decisions, we assessed cost-effectiveness using estimates of direct effects and spillover effects on prevalence. We calculated the incremental cost-effectiveness ratio (ICER) by dividing the difference in cost between arms by the difference in prevalent cases averted between arms. We included cases averted for both individuals within 500 m of any interventions and those with no intervention recipients within 500 m (direct-effect population) and at least one recipient within 500 m–3 km (spillover-effect population). Accounting for direct effects and spillover effects, the incremental cost-effectiveness ratios were US$144 (95% CI, $136–$153), $1,882 (95% CI, $1,679–$2,111) and $1,050 (95% CI $915–$1,231) for the chemoprevention, vector-control and combined interventions (Supplementary Table 11). Compared with the trial’s original incremental estimates of cost-effectiveness ratios, accounting for spillover effects increased cost-effectiveness by 11%, 30% and 42% for the chemoprevention, vector-control and combined interventions, respectively^37^.

### Test of contamination

**Extended Data Fig. 6:**
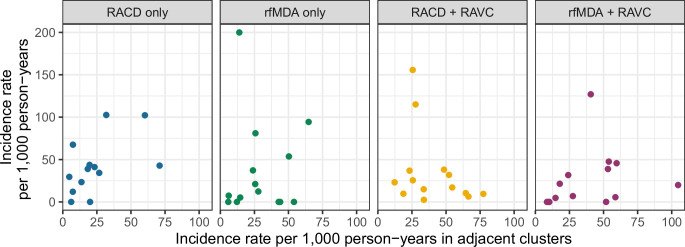
Cluster-level incidence by cluster-level incidence in adjacent clusters. Clusters were defined based on administrative areas, the randomization unit in the original trial.

**Extended Data Fig. 7:**
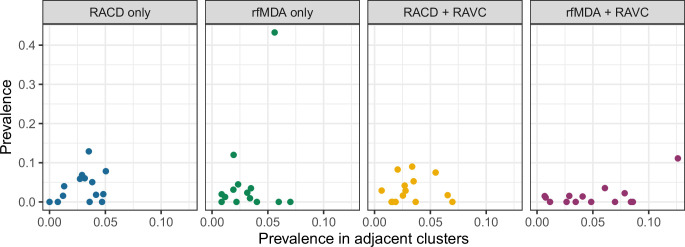
Cluster-level prevalence by cluster-level prevalence in adjacent clusters. Clusters were defined based on administrative areas, the randomization unit in the original trial.

Because the original studies did not include buffer zones between clusters, we tested for possible contamination between clusters, which might have biased original trial effect estimates towards the null. We used a likelihood ratio test to compare models of the effects of interventions that did or did not include the incidence or prevalence of adjacent study clusters. In the absence of contamination, we would expect that the incidence of a given cluster should not depend on that of adjacent clusters. There was no evidence that adjacent clusters’ incidence or prevalence were correlated with each other, suggesting that contamination did not occur (incidence *χ*^2^ = 0.540 and *P* = 0.462; prevalence *χ*^2^ = 0.0107 and *P* = 0.9178; Extended Data Figs. 6 and 7).

## Discussion

In this re-analysis of a cluster-randomized trial, we showed that a combined intervention of reactive focal chemoprevention with IRS reduced malaria incidence among non-recipients up to 1 km away and reduced malaria prevalence among non-recipients up to 3 km away. Evidence of spillover effects among non-recipients was strongest for the combined intervention, which was designed to reduce the parasite reservoir in both humans and mosquitos. When accounting for spillover effects, the cost-effectiveness of the combined intervention was 42% higher than the cost-effectiveness estimate that did not include spillover effects^37^.

Interventions that produce spillover effects yield greater population-health benefits at no additional cost. A prior analysis found that the combined intervention was highly cost-effective, but it did not account for possible spillover effects^37^. When accounting for spillover effects, interventions were 11–42% more cost-effective^37^. Given that malaria elimination requires substantially larger investments than does malaria control^22,38^, evidence about cost savings owing to spillover effects is critical to policy decisions about elimination strategies.

We found stronger evidence of spillover effects of the combined chemoprevention intervention over larger spatial scales than did two prior studies of targeted malaria interventions. In Kenya, a trial in a low-transmission area found no change in parasite prevalence within 500 m of serologically defined hotspots that received targeted larviciding, long-lasting insecticide-treated nets, IRS and focal mass drug administration^39^. In Zambia, an observational study in a high-transmission setting found that IRS targeted to subdistricts with higher malaria incidence and population density reduced parasite prevalence in sprayed and unsprayed households within target areas; it did not measure spillover effects outside of target areas^19^. The interventions in our study could have been more likely to produce spillover effects because they were delivered repeatedly in response to subsequent index cases. In this trial, each cluster received interventions up to 17 times; interventions were repeated annually over 3 years in the Zambia study and once in the Kenya trial. Further, it is possible that delivering interventions in response to new index cases can more effectively reduce transmission than can targeting interventions on the basis of an area’s incidence or seroprevalence.

For the chemoprevention intervention, there was no evidence of direct effects or spillover effects in primary analyses of malaria incidence; however, in secondary-outcome analyses, there was evidence of direct effects on seroprevalence and of spillover effects on prevalence. One potential explanation for this finding is that the reactive case detection intervention delivered artemether–lumefantrine and single-dose primaquine, whereas the chemoprevention intervention delivered only artemether–lumefantrine. It is possible that the inclusion of primaquine in the comparison arm attenuated the effect of chemoprevention towards the null, compared with the benefit that we would have observed if the same drugs were used in each arm^40^. However, this is unlikely given that the number of treated individuals was substantially higher in the rfMDA arms than in the RACD arms. Another possible explanation for our finding that there were impacts on prevalence but not incidence is that incidence analyses measured effects shortly after interventions, whereas prevalence analyses measured them at the end of the transmission season. Thus, our findings could indicate that reductions in local transmission accrued as additional rounds of chemoprevention interventions were delivered and population intervention coverage increased. This could be especially true for the chemoprevention intervention, because reductions in infectiousness of malaria cases are typically short-lived following treatment, especially in the absence of concurrent vector control^41^. Overall, these findings suggest that reactive, focal chemoprevention can more effectively reduce asymptomatic or subclinical infections among nearby non-intervention recipients than can RACD, particularly after repeated rounds.

For the vector-control intervention, there is a strong biological plausibility for direct effects and spillover effects of indoor residual spraying with pirimiphos-methyl. Further, female adult mosquitos were highly susceptible to pirimiphos-methyl in bioassays conducted in the trial, showing 100% mortality^24^. However, the primary analysis did not find direct effects, and the spillover-effect estimate included the null. In a 6-month follow-up period, we examined the longer-term effects of IRS, which resulted in spatiotemporal overlap between analytic cohorts (Supplementary Table 7). This overlap could have induced dependence between outcomes that was not fully accounted for by covariate adjustment, resulting in residual bias^30^. Analyses of current infection prevalence were not subject to concerns about cohort overlap and were suggestive of direct effects, but confidence intervals included the null and there was no evidence of spillover effects. Finally, our pre-specified subgroup analyses suggested that spillover effects of RAVC were driven by baseline transmission levels and environmental conditions: spillover effects on incidence were present in areas in which the baseline malaria incidence was <14 cases per 1,000 individuals, and when weather conditions favored mosquito breeding and survival (temperature < 31 °C; monthly rainfall < 27 mm).

The combined intervention seemed to have synergistic effects, reducing local transmission to intervention non-recipients through spillover effects on incidence, prevalence and seroprevalence. This could be because short-lived reductions in host infectiousness following chemotherapy can be sustained over time when coupled with the long-lasting reductions in mosquito populations caused by IRS. In effect, each intervention reduces the parasite reservoirs in hosts and vectors, and the combination of interventions slows the replenishment of parasite reservoirs^41^. This could explain why we found that spillover effects were larger for the prevalence of current infection, which captured effects at the end of the season, rather than the incidence, which captured short-term effects. Our findings are consistent with two recent studies that found evidence of potential synergistic community-level effects when combining community-wide chemoprevention or seasonal malaria chemoprevention with IRS in high-transmission settings^42,43^. Our results are also consistent with a modeling study that estimated that the joint effect of chemoprevention and IRS was more than 1.5 times higher than effects of intervention alone in low-transmission settings^41^. Taken together, our results suggest that the combined intervention could be particularly effective as a reactive intervention or outbreak response in low-transmission settings approaching elimination or possibly following introduction of cases after elimination has been achieved.

Subgroup analyses suggested that there were spillover effects among men but not women, particularly for the chemoprevention intervention. This could be because men had a higher malaria risk overall, owing to their greater likelihood of undertaking travel and work activities outside the home. Sixty percent of index cases in the study were in men, and 12% of men were of Zambian nationality, as opposed to 7% of women^24^. A prior study in Namibia found that traveling was associated with increased malaria risk among men but not women, and among non-travelers, malaria risk was almost twice as high among men as among women^44^. This could be because men are less likely to use preventive measures and might spend more time outside at night.

Our findings shed light on the mechanism through which these targeted interventions work in time and space. For the interventions that produced spillover effects, we found that spillover-effect sizes were generally similar to or stronger than direct-effect sizes. It is possible that, during the time between index-case detection and intervention delivery (median, 13–14 days)^24^, transmission occurred to nearby intervention recipients. Thus, the interventions might not have been rapid enough to reduce malaria among recipients, but could have prevented onward transmission to non-recipients farther from index cases. In addition, our finding that spillover effects on prevalence were stronger at shorter distances to the interventions suggests that the majority of the spillover effect occurred within 1 km of index cases.

Our analysis used data from a randomized trial that achieved high intervention coverage (>85% of the target population in all arms) and delivered focal interventions up to 17 times per cluster within a 1-year period^24^. In a programmatic setting, it might be difficult to achieve this level of intervention coverage, and it is possible that spillover effects would be smaller or absent at lower coverage levels. We were not able to assess effect modification by intervention coverage, because coverage was high across study clusters. Another prior trial of reactive, focal MDA that had lower intervention coverage failed to detect effects on incidence in cluster-level analyses^45^.

This study was conducted in a low-transmission region, with a typical annual malaria incidence of approximately 15 cases per 1,000 people in the years prior to the trial, and 41 cases per 1,000 people in the standard-of-care control arm (RACD only) during the year in which the trial began. The spillover effects that we estimated in this study might not be generalizable to settings with a different transmission intensity or places where transmission is associated with demographic risks, such as occupation or travel, and does not occur at the household level.

Our study was subject to several limitations. First, owing to rare outcomes, precision was low in some analyses and might have increased the chance of type II error. In particular, analyses of direct effects on incidence used a smaller sample size than did spillover-effects analyses and were likely underpowered; this could explain why we unexpectedly found spillover effects for the chemoprevention and combined interventions, but not direct effects in the incidence analysis. However, we did find direct effects of these interventions in studies of seroprevalence, which capture recent infections and have higher statistical power. We had originally planned to do a meta-analysis of individual participants incorporating data from the present trials and two other trials in Eswatini and Zambia to increase precision; however, at those sites, geocoding of participants was not sufficient to allow for the planned spillover analyses. Second, when constructing analytic cohorts, household relocation after baseline could have resulted in misclassification of households to target areas or spillover zones. Third, malaria incidence for the year prior to the trial was not included in the trial’s restricted randomization, and as a result, malaria incidence in the year before the trial was higher in intervention clusters than in RACD clusters. Our analyses accounted for this by including 2016 cluster malaria incidence in the covariate adjustment set, consistent with the original statistical analysis of the trial. If adjustment did not fully account for differing baseline transmission intensities between study arms, we would expect that effects were underestimated. Fourth, in serologic analyses, individuals in whom the Etramp5.Ag1 biomarker was detected might have had infections that preceded delivery of interventions in their area, which could have biased estimates towards the null. Finally, incidence analyses could not fully rely on randomization-based inference owing to cohort overlap (Fig. 1); it is possible that covariate adjustment did not fully account for imbalances between arms. Overlap between cohorts could have attenuated effect estimates towards the null, particularly for the vector-control intervention arms, which had a greater degree of overlap. Our prevalence analysis, which was not subject to these limitations, also found a spillover effect of the combined intervention, which was stronger than that in the incidence analysis; this internal consistency lends credibility to our conclusion that the combined intervention produced spillover effects. In future studies, using a ring-trial design to test focal interventions could improve baseline balance, increase precision and minimize overlap between target areas^26^.

Despite these limitations, the internal consistency between the findings of this analysis and the original trial analysis, which each used different data structures and statistical methods, supports the validity of our findings. Estimates of total effects in this analysis, which were pooled across intervention recipients and non-recipients, were consistent overall with those of the original trial, which included all individuals in study clusters (intervention recipients and non-recipients)^24,46^. Additional strengths include pre-specification of spillover-analysis methods and use of individual-level, spatially indexed data to measure spillovers.

In conclusion, we found that reactive, focal malaria interventions that combined chemoprevention and IRS reduced malaria among non-intervention recipients up to 3 km from index cases. Accounting for spillover effects led to meaningful increases in the cost-effectiveness of the intervention. These findings, together with the original cluster-level analysis of the trial, suggest that combined reactive, focal interventions are an effective strategy in low-transmission, malaria-elimination settings.

## Methods

### Analysis overview

In this study, data were analyzed from a previously completed cluster-randomized trial of focal malaria interventions conducted in Zambezi region of Namibia from 1 January to 31 December 2017 (NCT02610400)^24,47^. The analysis plan was pre-specified and is available at https://osf.io/s8ay4/. Deviations from the pre-analysis plan are in the Supplementary Information.

### Study population

The Zambezi region has seasonal malaria transmission that peaks between January and June. *Pf* is the dominant species, and annual *Pf* incidence was less than 15 cases per 1,000 people from 2010 to 2015. In 2016, the incidence was 32.5 cases per 1,000 people following an outbreak^25^. In 2015, prevalence measured by loop-mediated isothermal amplification was 2.2%^48^. At the study site, the Namibia Ministry of Health and Social Services routinely delivered case management and annual preseason household IRS with dichlorodiphenyltrichloroethane (DDT), with the exception of a small number of structures that were sprayed with deltamethrin. In addition, they offered reactive case detection (RACD) within 500 m of confirmed malaria cases, which included testing with rapid diagnostic tests and treatment with artemether–lumefantrine and single-dose primaquine for those who tested positive.

### Trial eligibility

The trial defined clusters on the basis of census enumeration areas that were within the catchment area of study healthcare facilities. Enumeration areas were eligible for inclusion in the trial if they (1) were located in the catchment areas of 11 health facilities, (2) had complete incidence data from 2012–13 and (3) had at least one incident case during the trial; 102 enumeration areas were assessed for eligibility, and 46 had no incident malaria cases from 2012–14 or incomplete incidence data and were excluded from the study population. Because of the limited number of eligible enumeration areas, the study did not include buffer zones between clusters.

### Informed consent

In the original trial, written informed consent was obtained from individual participants for rfMDA or RACD, and from heads of households (≥18 years of age) for RAVC. A parent or guardian was required to provide written informed consent for children younger than 18 years receiving rfMDA or RACD, and written assent for receiving these interventions was also obtained from children aged 12–17 years.

### Randomization and blinding

In the trial, which had a two-by-two factorial design 56 clusters were randomly allocated to study arms: RACD only; rfMDA only; RACD and RAVC; or rfMDA and RAVC. The trial used restricted randomization with the following criteria: mean annual incidence in 2013 and 2014, population size, population density and mean distance from the household to a healthcare facility. Incidence data for 2013 and 2014 instead of 2015 and 2016 (the years immediately before the trial) were used because the data for 2015 were not available, and transmission was unusually high in 2016 owing to an outbreak. The study statistician randomly generated 100,000 assignments meeting restriction criteria. The Namibia Ministry of Health and Social Services randomly selected the allocation from these assignments. It was not practical to blind study participants or field staff to intervention assignment, but laboratory analyses and primary statistical analyses were blinded.

### Interventions

Field staff delivered interventions in response to passively detected malaria index cases that were confirmed by rapid diagnostic tests or microscopy if the case had resided in the study cluster at least one night in the prior 4 weeks. The trial delivered interventions in ‘target areas’ within approximately 500 m of confirmed malaria cases detected through passive surveillance. In the RACD arms, individuals were eligible to receive rapid diagnostic tests, and individuals who tested positive were eligible for treatment with artemether–lumefantrine and single-dose primaquine (Coartem, Novartis Pharmaceuticals, or Komefan 140, Mylan Laboratories). In the rfMDA arms, individuals were eligible for presumptive treatment with artemether–lumefantrine. In the RAVC arms, households were eligible for IRS with pirimiphos-methyl (Actellic 300CS, Syngenta), which has been shown to reduce entomologic transmission indicators, such as *Anopheles* density, parity rate, entomological inoculation rate, vector longevity, biting rates and sporozoite infections^49–51^. In all arms, study teams delivered interventions within 500 m of a clinical malaria case. On average, the median number of days between reporting of an index case and intervention delivery within clusters was 16 (s.d. = 6). If the study team was not able to deliver an intervention within 5 weeks of index case detection owing to a high case load, no intervention was given. RACD and rfMDA interventions were delivered to at least 25 people within target areas, and RAVC was delivered to at least seven households within target areas. More than 80% of eligible confirmed malaria cases received interventions, and more than 85% of eligible intervention recipients were covered by interventions^24^. Field staff did not offer repeat interventions in response to subsequent index cases within 5 weeks for rfMDA and RACD and within the same malaria season for RAVC. Field staff recorded the household geocoordinates of the index case and intervention recipients. Additional details about the interventions were previously published^24,47^.

### Procedures

Prior to randomization, field staff conducted a geographic census and recorded the latitude and longitude of all households in the study area. During the trial, trial staff extracted data on confirmed incident malaria cases and travel history from the rapid reporting system. At the end of malaria season between May and August 2017, the study team collected an endline cross-sectional survey to measure infection prevalence. Field staff collected dried blood spots on filter paper (Whatman 3 Corporation) by finger prick, and qPCR was performed targeting the acidic terminal sequence of the *var* gene^52^. Field staff also collected 250 ml of whole blood in BD Microtainer tubes with EDTA additive (Becton, Dickinson and Corporation) for serological analyses. Luminex assays were performed on human plasma to detect malaria antigens using previously described procedures^46,53^. Field staff recorded the geocoordinates of all households in the cross-sectional survey.

### Analysis overview

Here, we separately estimated effects among intervention recipients and non-recipients to estimate direct effects and spillover effects. We estimated the effects of the chemoprevention intervention (rfMDA versus RACD; *n* = 28 clusters versus *n* = 28 clusters), the vector-control intervention (RAVC versus no RAVC; *n* = 28 clusters versus *n* = 28 clusters) and combined interventions (rfMDA and RAVC versus RACD only; *n* = 14 clusters versus *n* = 14 clusters), consistent with the original trial^24^ (Supplementary Table 1). Because compliance was high, we compared outcomes between arms using randomized treatment assignments. The pre-specified outcomes were standard malariometric measures including clinical incidence of malaria detected at health facilities (primary outcome), as well as cross-sectional prevalence of current infection and seroprevalence measured in an endline survey (secondary outcomes).

### Incidence analyses

To capture the person-time and area in which we expected each intervention to influence incident malaria infections, we created analytic cohorts in space and time around each index case that triggered an intervention. The primary analysis used a 1-km radius around each index case, because the majority of *Anopheles* mosquito movement occurs within <1 km^34^.

### Construction of analytic cohorts for incidence analysis

To construct cohorts, we matched index cases and intervention recipients to individuals recorded in the baseline census using household geocoordinates, age and sex. We required that geocoordinates be <100 m apart to allow for small deviations in the location of geocoordinate recordings. We excluded 32 cohorts from the analysis for which it was not possible to merge intervention recipient geocoordinates with index data geocoordinates. Clusters were contiguous with no buffer zones between them, so to capture potential dependencies across study clusters, we allowed cohorts to include individuals assigned to an adjacent cluster with a different treatment assignment from the triggering index case if it was within 1 km of an index case.

We pre-specified the cohort follow-up length on the basis of the period in which we expected each intervention to reduce malaria among intervention recipients (direct effects) and non-recipients (spillover effects). Day 0 for each cohort was the date of intervention delivery. For comparisons of rfMDA and RACD interventions, the follow-up period for direct effects was 0 to 35 days, the length of intrinsic incubation period for *Pf* malaria^27^. This is the period of time in which we would expect the intervention to interrupt the parasite life cycle in treated, infected individuals, and in turn, prevent symptoms and/or infectiousness. The follow-up period for spillover effects was 21 to 56 days; the 3-week lag period allowed for gametocyte clearance in the treated individual, sporozoite development in mosquitos and development of detectable merozoites in humans. For RAVC interventions, the follow-up period for direct effects was 6 months because IRS can remain effective for an entire transmission season^28^. The follow-up period for spillover effects was from day 17 to 6 months. A mosquito bite could hypothetically be prevented on the day of intervention, so the earliest secondary case could occur after sporozoite development in mosquitos (minimum 10 days), and development of detectable merozoites in humans (minimum 7 days).

### Statistical models for incidence

We conducted statistical analyses using R version 4.1.0. To compare incidence between arms, we used hierarchical TMLE, a doubly-robust, semi-parametric approach that is appropriate for cluster-level exposures^29^. TMLE estimates both an outcome regression and a propensity score (the probability of treatment calculated on the basis of the covariates) and updates the initial parameter estimate using information in the propensity score. Compared with other parametric models for clustered data (for example, mixed effects models and generalized estimating equations), hierarchical TMLE imposes fewer assumptions and can be more efficient for randomized trials^54^. We fit propensity-score models at the cohort level because interventions were delivered to cohorts. Within study clusters and cohorts, we expected individuals’ outcomes to be correlated owing to interventions, social interactions and local environmental factors. We fit two types of outcome models that accounted for statistical dependence in different ways^54^. Cohort-level models allowed for statistical dependence between individuals in the same cohort without making any assumptions about the nature of the dependency. Individual-level models assumed that cluster-level and individual-level covariates removed any dependence between outcomes of individuals in nearby geographic areas^54^.

Because the incidence analyses used analytic cohorts, the unit of analysis differed from the unit of randomization (clusters), and there were differences in some baseline characteristics between clusters and analytic cohorts (Supplementary Table 2). Additionally, although cohort-intervention assignments were the same as the intervention assignments of their respective clusters, some cohorts overlapped with others. As such, we could not fully rely on randomization-based inference. Furthermore, in the original trial, malaria incidence in the year before the trial was not balanced between study arms^24^. Thus, we adjusted for covariates, including cluster intervention assignments.

Propensity-score models adjusted for the following baseline variables: cluster-level IRS coverage, malaria incidence, median monthly rainfall, median enhanced vegetative index, median daytime land surface temperature in the season prior to the trial, population size and median elevation. Outcome models adjusted for individual- and cluster-level covariates. Individual-level covariates included sex, age, calendar month of intervention, distance from an individual’s residence to the residence of the index case that triggered an intervention, the number of interventions that an individual previously received, the number of previous intervention recipients within 0.5, 1, 2 and 3 km of the individual’s residence (from the start of the trial to the start of the cohort’s observation period) and the population size within 0.5, 1, 2 and 3 km of the individual’s residence. Cluster-level covariates included those in the propensity-score models as well as mean distance to the nearest neighboring household, mean distance to the nearest healthcare facility and mean time from index case detection to intervention (see further details in the Supplementary Information).

We fit outcome and propensity-score models that adjusted for covariates using an ensemble machine-learning algorithm^55^. We used the SuperLearner with tenfold cross-validation to construct a convex combination of algorithm predictions that minimized the cross-validated mean squared error (SuperLearner R package version 2.0–28.1; sl3 R package version 1.4.4). This ensemble approach trained each algorithm on different random subsets of the data, which can reduce the influence of covariates that have a strong impact on a particular model. Using a weighted average of predictions from candidate learners can also reduce the impact of individual covariates with an outsize influence in a single model and increase model robustness and stability. Using V-fold cross-validation also helps to minimize overfitting. For propensity-score models, learners included generalized linear models, least absolute shrinkage and selection operator (LASSO)^56^ and elastic net regression^57^ (glmnet R package version 4.1–2). For outcome models, we used the same learners as well as extreme gradient boosting (xgboost R package version 1.4.1.1)^58^. The extreme gradient boosting algorithm included 20 fitting iterations with a learning rate of 0.3 and a maximum tree depth of 6. To minimize empirical positivity violations, if there were fewer than 20 observations per covariate, we included separate generalized linear model learners with submodels adjusting for two covariates at a time; each learner adjusted for cluster-level intervention assignment and a single additional covariate that was correlated with the outcome. We performed 10-fold cross-validation using the mean squared error as the loss function at either the individual or cohort level^54^. Validation samples were constructed from randomly sampled individuals or cohorts. We separately fit individual- and cohort-level models and then chose the outcome model with the smaller cross-validated mean squared error. We chose the mean squared error as the loss function because it minimizes outlier predictions with very large errors. For comparisons of rfMDA and RAVC versus RACD, which had rare outcomes and a smaller sample size, we used 30-fold cross-validation.

The particular set of covariates included in each model was data-driven and based on (1) initial screening for covariates associated with the outcome (likelihood ratio test *P* < 0.2)^59^ and (2) covariate selection within candidate learners included in the SuperLearner (for example, feature importance ranking or regularization). Further, ensemble methods create weighted average predictions from learners using different covariate sets to minimize overfitting. Thus, adjustment covariates varied between models.

### Adjusting standard errors for cohort overlap

We adjusted the standard errors to account for potential correlation caused by overlap between some cohorts using a model of cohort-level influence curves that is analogous to the variance–covariance models used in cross-random effects models^60,61^. Specifically, we fit the model:

1 $${D}_{i}\times {D}_{j} \sim d(i,j)+t(i,j)+C$$

where *D*_*i*_ × *D*_*j*_ is the product of influence curves of cohorts *i* and *j*, *d*(*i*, *j*) is the distance between the location of the index case that triggered the intervention in each cohort, *t*(*i*, *j*) is the start date of the intervention in each cohort and *C* is the cluster-level intervention assignment^62^. Adjustment for intervention assignment accounted for correlation due to shared exposure to the intervention or receipt of the intervention. For cohorts with no overlap, we set *D*_*i*_ × *D*_*j*_ to zero. The regression was implemented with a simplified SuperLearner library including the generalized linear models and LASSO^56^. We calculated the variance accounting for outcome dependence as follows:

$$\mathrm{var}(\hat{\psi}-\psi )=\mathrm{var}\left(\frac{1}{n}\mathop{\sum }\limits_{i=1}^{n}{D}_{i}\right)=\frac{1}{{n}^{2}}\left(\mathop{\sum }\limits_{i=1}^{n}\mathrm{var}\left({D}_{i}\right)+2\sum _{i < j}\mathrm{cov}({D}_{i},{D}_{j})\right)$$

where $\hat{\psi }$ is the estimator, $\psi$ is the estimand and *n* is the number of cohorts.

### Subgroup analyses for incidence

Subgroup analyses of spillover effects repeated analyses within each subgroup stratum, and we drew inferences about effect heterogeneity by comparing the point estimates from a given stratum with the confidence interval for other strata.

### Sensitivity analyses for incidence

We conducted several sensitivity analyses for the primary analysis. (1) We constructed spillover cohorts with 2- and 3-km radii around index cases to capture mosquito dispersal over longer distances^34,35^. (2) We used alternative follow-up period lengths in which intervention effects might have been stronger (rfMDA and RACD direct effects, days 0–21; spillover effects, days 21–42; RAVC direct effects, days 0–7; spillover effects, days 17–90). (3) Because <3% of intervention recipients resided outside of target areas (>500 m from index cases), we repeated direct-effects analyses including all individuals receiving treatment regardless of distance to the index case. (4) To account for overlap in analytic cohorts (Supplementary Table 3), which may have resulted in statistical dependence between outcomes, we excluded overlapping cohorts from the analysis.

### Prevalence analyses

To capture effects of cumulative interventions at the end of the malaria-transmission season, we estimated effects on prevalence using data from a cross-sectional survey. Direct-effects analyses included individuals who had any intervention recipients within 500 m of their residence, spillover-effects analyses included individuals with no intervention recipients within 500 m and at least one recipient within 500 m–3 km and total-effects analyses included individuals with at least one intervention recipient within 3 km.

### Statistical models for prevalence

Prevalence analyses used data from the cross-sectional survey conducted at the end of the 2017 malaria-transmission season. We used TMLE^63^ with individual-level data with the same learners included in incidence analyses (tmle3 R package version 0.2.0). We accounted for correlation within enumeration-area-level clusters using cluster-level influence-curve-based standard errors. The covariate set included the same cluster-level covariates included in incidence analyses and the following individual-level covariates: age, sex, occupation, recent travel, whether the household slept under a bed net, whether the individual slept outdoors in the past two weeks, the total population, the number of intervention recipients, the number of intervention recipients in the same study arm, the number of intervention recipients in a different study arm and the proportion of intervention recipients of the same treatment within 500 m, 1 km, 2 km and 3 km of sampled individuals (see further details in the Supplementary Information). We screened for covariates using the same approach described for incidence analyses.

In both incidence and prevalence analyses, we excluded any categorical covariates with less than 5% prevalence to avoid positivity violations. To minimize empirical positivity violations^64^, we only fit models if the number of outcome events per variable was ≥10, and we only fit adjusted models if the number of observations per strata was ≥30 (ref. ^65^).

### Cost-effectiveness analysis

We estimated ICERs by incorporating direct effects and spillover effects. To facilitate comparison with the original cost-effectiveness estimates for the trial^37^, we used estimated effects on prevalence measured by qPCR. ICERs estimated using incidence would not be directly comparable between this study and the original trial because we used a cohort-level analysis and the original trial used a cluster-level analysis. We used previously published estimates of total intervention costs in 2017 in US dollars^37^. To obtain the number of prevalent cases averted, we multiplied the difference in prevalence between arms among intervention recipients and non-recipients from models of the total effect by the estimated population size included in total effects analyses. We calculated the ICER by dividing the difference in cost between arms by the difference in prevalent cases averted between arms among individuals who were located within 500 m of any intervention recipients and individuals who resided within 500 m to 3 km of interventions. To obtain 95% confidence intervals, because we used total intervention cost measured without error, we applied the ICER formula to the lower and upper bounds of the 95% confidence interval for the total effect.

### Test of contamination

We tested for possible contamination between clusters since the trial did not include buffer zones. We used a log-linear model to regress incidence and prevalence in each study cluster on an indicator for randomized treatment assignment as well as the incidence in adjacent study clusters. In the absence of contamination, we would expect that the incidence of a given cluster should not depend on that of adjacent clusters. We used a likelihood ratio test to compare models with and without independent variables for the cumulative incidence or prevalence in adjacent study clusters.

### Ethics statement

The trial protocol was approved by the Namibia Ministry of Health and Social Services (17/3/3) and the Institutional Review Boards at the University of California San Francisco (15–17422) and London School of Hygiene & Tropical Medicine (10411). The protocol for this analysis was approved by the Stanford University Institutional Review Board (60708).

### Inclusion and ethics

This study analyzed data that were collected in a cluster-randomized controlled trial in the Zambezi region of Namibia. The data are owned by the original investigators that collected the data, and the investigators have also made the data publicly available. Researchers from Namibia were included in the process of obtaining funding, developing the statistical-analysis plan, interpreting study findings and writing this manuscript. Analysis code has been published with the manuscript to promote transparency and extension of our research by local and global investigators.

### Reporting summary

Further information on research design is available in the Nature Portfolio Reporting Summary linked to this article.

## Online content

Any methods, additional references, Nature Portfolio reporting summaries, source data, extended data, supplementary information, acknowledgements, peer review information; details of author contributions and competing interests; and statements of data and code availability are available at 10.1038/s41591-024-03134-z.

## Supplementary information

Supplementary Information Supplementary Tables 1–11

Reporting Summary

## Data Availability

Data from the original trial are available at https://clinepidb.org/ce/app/workspace/analyses/DS_f559aee789 or from M.H. (michelle.hsiang@ucsf.edu). All data requests that comply with limitations contained in the informed consent form signed by the participants of the trial will be granted access.

## Code availability

Replication scripts are available from Zenodo at 10.5281/zenodo.11410094 (ref. ^66^).
