# Supplementary material for: Extension of efficacy range for targeted malaria-elimination interventions due to spillover effects
Source: Nat Med. 2024 Jul 4;30(10):2813–20. doi: 10.1038/s41591-024-03134-z (PMC11483210; doi:10.1038/s41591-024-03134-z)
Supplement: Supplementary file 1 — Supplementary Tables 1–11 [file 41591_2024_3134_MOESM1_ESM.pdf]

nature medicine

Supplementary information https://doi.org/10.1038/s41591-024-03134-z

Extension of efficacy range for targeted malaria-elimination interventions due to spillover effects

In the format provided by the authors and unedited

Supporting Information to "Extension of efficacy range for targeted malaria elimination interventions due to spill-over effects"

Supplementary Table 1. Two-by-two factorial study design of reactive focal interventions . 2

Supplementary Table 2. Baseline characteristics in study clusters and analytic cohorts 3

Supplementary Table 3. Baseline characteristics among intervention recipients and non-recipients. 4

Supplementary Table 4. Direct effect, spillover effect, and total effect estimates on cumulative incidence of malaria infection 5

Supplementary Table 5. Range above and below median value in each enumeration area for subgroup variables 6

Supplementary Table 6. Number of individuals and cohorts included in sensitivity analyses 7

Supplementary Table 7. Percentage of cohorts overlapping with other cohorts 8

Supplementary Table 8. Direct effect, spillover effect, and total effect estimates on malaria prevalence measured by qPCR 9

Supplementary Table 9. Direct effect, spillover effect, and total effect estimates on household-level malaria prevalence of measured by qPCR 10

Supplementary Table 10. Direct effect, spillover effect, and total effect estimates on Etramp5.Ag1 seroprevalence 11

Supplementary Table 11. Cost-effectiveness analysis 12

		**Chemoprevention intervention**	
		**Reactive case detection only** (28 clusters)	**Reactive focal mass drug administration** (28 clusters)
**Vector control intervention**	**No reactive focal vector control** (28 clusters)	A. Reactive case detection only **(RACD)** (14 clusters)	B. Reactive focal mass drug administration only **(rfMDA)** (14 clusters)
	**Reactive focal vector control** (28 clusters)	C. Reactive case detection plus reactive focal vector control **(RACD + RAVC)** (14 clusters)	D. Reactive focal mass drug administration plus reactive focal vector control **(rfMDA + RAVC)** (14 clusters)

Supplementary Table 1. Two-by-two factorial study design of reactive focal interventions
Reactive case detection (RACD) involved administering rapid diagnostic tests for malaria to individuals living within a 500-m radius of an index case and treating individuals who tested positive with artemether-lumefantrine and single-dose primaquine. Reactive focal mass drug administration (rfMDA) involved presumptively treating individuals living within a 500-m radius of an index case with artemether-lumefantrine, without testing for malaria beforehand. Reactive focal vector control (RAVC) involved spraying the long-lasting insecticide, pirimiphos-methyl, to the interior walls of households located within a seven-household radius of an index case. The effectiveness of three interventions were compared to three respective controls: (1) rfMDA versus RACD (B and D vs A and C); (2) RAVC versus no RAVC (C and D vs A and B); and (3) rfMDA plus RAVC versus a RACD only (D vs A). Reproduced from Hsiang et al. 2020 *Lancet* with permission.

	Study Clusters		Analytic Cohorts	
**Chemoprevention intervention**	**RACD**	**rfMDA**	**RACD**	**rfMDA**
**Population characteristics**				
Mean cluster/cohort population size (SE)	360 (24)	318 (22)	264 (9)	259 (12)
Malaria incidence per 1,000 in 2016 (SE)	28.5 (5.8)	36.5 (12.5)	29.0 (0.11)	42.6 (0.34)
Pre-season indoor residual spray coverage 2016 (SE)	77.22 (4.03)	76.55 (4.58)	77.0 (0.10)	80.6 (0.11)
Distance to nearest healthcare facility (km) (SE)	5.6 (0.8)	6.2 (1.0)	4.9 (0.02)	6.7 (0.03)
**Ecological factors (range)**				
Median monthly rainfall November 2016-April 2017 (mm)	23.7 (18.4, 26.7)	23.3 (18.4, 26.7)	23.7 (18.4, 26.7)	23.5 (18.4, 26.7)
Median enhanced vegetative index January 2017-July 2017	0.15 (0.09, 0.31)	0.15 (0.09, 0.27)	0.15 (0.09, 0.31)	0.15 (0.09, 0.27)
Median elevation (m)	522 (387, 1021)	560 (412, 1124)	522 (387, 1021)	535 (412, 1124)
Median daytime land surface temperature (C)	30.6 (28.9, 33.4)	31.4 (28.6, 32.5)	30.5 (28.9, 33.4)	31.1 (28.6, 32.5)
**Vector control intervention**	**No RAVC**	**RAVC**	**No RAVC**	**RAVC**
**Population characteristics**				
Mean cluster/cohort population size (SE)	339 (22)	338 (24)	249 (9)	274 (11)
Malaria incidence per 1,000 in 2016 (SE)	26.1 (6.4)	38.9 (12.1)	28.7 (0.16)	41.4 (0.29)
Pre-season indoor residual spray coverage 2016 (SE)	77.98 (4.64)	75.82 (3.99)	78.6 (0.11)	78.8 (0.09)
Distance to nearest healthcare facility (km) (SE)	4.9 (0.8)	6.8 (0.9)	4.4 (0.02)	6.9 (0.02)
**Ecological factors (range)**				
Median monthly rainfall November 2016-April 2017 (mm)	23.5 (18.4, 26.7)	23.7 (18.4, 26.7)	23.7 (18.4, 26.7)	23.7 (18.4, 26.7)
Median enhanced vegetative index January 2017-July 2017	0.15 (0.09, 0.22)	0.15 (0.09, 0.31)	0.15 (0.09, 0.22)	0.15 (0.09, 0.31)
Median elevation (m)	535 (398, 1124)	544 (387, 1021)	527 (398, 1124)	547 (387, 1021)
Median daytime land surface temperature (C)	31.1 (28.6, 33.4)	31.1 (28.7, 32.5)	30.7 (28.6, 33.4)	30.8 (28.7, 32.5)
**Combined intervention**	**RACD only**	**rfMDA+RAVC**	**RACD only**	**rfMDA+RAVC**
**Population characteristics**				
Mean cluster/cohort population size (SE)	350 (25)	308 (26)	282 (13)	305 (19)
Malaria incidence per 1,000 in 2016 (SE)	27.3 (8.1)	48.1 (22.9)	27.3 (0.16)	52.4 (0.55)
Pre-season indoor residual spray coverage 2016 (SE)	84.25 (5.66)	80.93 (5.82)	82.9 (0.13)	86.4 (0.12)
Distance to nearest healthcare facility (km) (SE)	3.9 (0.8)	6.6 (1.5)	3.2 (0.02)	7.3 (0.04)
**Ecological factors (range)**				
Median monthly rainfall November 2016-April 2017 (mm)	23.7 (18.4, 26.7)	23.4 (18.4, 26.7)	23.7 (18.4, 26.7)	23.7 (18.4, 26.7)
Median enhanced vegetative index January 2017-July 2017	0.15 (0.10, 0.21)	0.15 (0.09, 0.27)	0.15 (0.10, 0.21)	0.15 (0.09, 0.27)
Median elevation (m)	522 (398, 921)	558 (412, 984)	522 (398, 921)	576 (412, 984)
Median daytime land surface temperature (C)	30.7 (28.9, 33.4)	31.4 (28.7, 32.5)	30.6 (28.9, 33.4)	31.1 (28.7, 32.5)

Supplementary Table 2. Baseline characteristics in study clusters and analytic cohorts

Includes 55 study clusters that were randomized in the original trial and had at least one index case during follow-up and 310 analytic cohorts used in the analysis of spillover effects and direct effects.

	Intervention recipients		Non-recipients	
**Chemoprevention intervention**	**RACD**	**rfMDA**	**RACD**	**rfMDA**
**Population characteristics**				
Number of cohorts	161	149	161	149
Mean cohort population size (SE)	26 (1)	27 (1)	238 (9)	232 (12)
Mean cluster population size (SE)	389.6 (1.94)	346.4 (1.96)	379.4 (0.63)	355.8 (0.59)
Malaria incidence per 1,000 in 2016 (SE)	27.0 (0.37)	55.8 (1.26)	29.2 (0.12)	41.0 (0.35)
Pre-season indoor residual spray coverage 2016 (SE)	76.3 (0.32)	77.1 (0.36)	77.1 (0.10)	81.0 (0.12)
Distance to nearest healthcare facility (km) (SE)	5.2 (0.06)	6.7 (0.08)	4.9 (0.02)	6.7 (0.03)
**Ecological factors (range)**				
Median monthly rainfall November 2016-April 2017 (mm)	23.7 (18.4, 26.7)	23.5 (18.4, 26.7)	23.7 (18.4, 26.7)	23.5 (18.4, 26.7)
Median enhanced vegetative index January 2017-July 2017	0.15 (0.09, 0.31)	0.15 (0.09, 0.27)	0.15 (0.09, 0.31)	0.15 (0.09, 0.27)
Median elevation (m)	522 (387, 1021)	541 (412, 1124)	522 (387, 1021)	535 (412, 1124)
Median daytime land surface temperature (C)	30.5 (28.9, 33.4)	31.1 (28.6, 32.5)	30.5 (28.9, 33.4)	31.1 (28.6, 32.5)
**Vector control intervention**	**No RAVC**	**RAVC**	**No RAVC**	**RAVC**
**Population characteristics**				
Number of cohorts	152	158	152	158
Mean cohort population size (SE)	26 (1)	27 (1)	223 (9)	247 (11)
Mean cluster population size (SE)	358.9 (2.01)	376.9 (1.94)	354.0 (0.58)	380.5 (0.63)
Malaria incidence per 1,000 in 2016 (SE)	31.9 (0.60)	50.0 (1.15)	28.3 (0.17)	40.4 (0.29)
Pre-season indoor residual spray coverage 2016 (SE)	77.9 (0.37)	75.6 (0.31)	78.6 (0.12)	79.2 (0.10)
Distance to nearest healthcare facility (km) (SE)	5.0 (0.06)	6.7 (0.07)	4.4 (0.02)	6.9 (0.02)
**Ecological factors (range)**				
Median monthly rainfall November 2016-April 2017 (mm)	23.5 (18.4, 26.7)	23.7 (18.4, 26.7)	23.7 (18.4, 26.7)	23.7 (18.4, 26.7)
Median enhanced vegetative index January 2017-July 2017	0.15 (0.09, 0.22)	0.15 (0.09, 0.31)	0.15 (0.09, 0.22)	0.15 (0.09, 0.31)
Median elevation (m)	527 (398, 1124)	547 (387, 1021)	527 (398, 1124)	547 (387, 1021)
Median daytime land surface temperature (C)	30.7 (28.6, 33.4)	30.8 (28.7, 32.5)	30.7 (28.6, 33.4)	30.8 (28.7, 32.5)
**Combined intervention**	**RACD only**	**rfMDA+RAVC**	**RACD only**	**rfMDA+RAVC**
**Population characteristics**				
Number of cohorts	73	70	73	70
Mean cohort population size (SE)	26 (1)	29 (1)	256 (13)	276 (19)
Mean cluster population size (SE)	353.0 (2.05)	328.0 (1.89)	349.5 (0.61)	352.8 (0.63)
Malaria incidence per 1,000 in 2016 (SE)	26.6 (0.56)	75.4 (2.25)	27.3 (0.16)	50.0 (0.55)
Pre-season indoor residual spray coverage 2016 (SE)	83.6 (0.43)	81.5 (0.42)	82.8 (0.14)	86.9 (0.12)
Distance to nearest healthcare facility (km) (SE)	3.5 (0.06)	6.9 (0.12)	3.2 (0.02)	7.3 (0.04)
**Ecological factors (range)**				
Median monthly rainfall November 2016-April 2017 (mm)	23.7 (18.4, 26.7)	23.7 (18.4, 26.7)	23.7 (18.4, 26.7)	23.7 (18.4, 26.7)
Median enhanced vegetative index January 2017-July 2017	0.15 (0.10, 0.21)	0.15 (0.09, 0.27)	0.15 (0.10, 0.21)	0.15 (0.09, 0.27)
Median elevation (m)	522 (398, 921)	576 (412, 984)	522 (398, 921)	677 (412, 984)
Median daytime land surface temperature (C)	30.7 (28.9, 33.4)	31.1 (28.7, 32.5)	30.6 (28.9, 33.4)	31.1 (28.7, 32.5)

Supplementary Table 3. Baseline characteristics among intervention recipients and non-recipients.
Includes data from intervention recipients in target areas located within 500m of an index case and non-recipients up to 1km from an index case that triggered interventions.

			Incidence proportion		Incidence ratio (95% CI)		
	N cohorts	N	Intervention arm	Reference arm	Unadjusted	Adjusted	Adjusted, CI adjusted for cohort overlap
**Chemoprevention intervention** (rfMDA vs. RACD)							
Direct effect	310	8,252	3.4	6.5	0.53 (0.25, 1.11)	0.40 (0.11, 1.48)	0.40 (0.10, 1.56)
Spillover effect	310	72,830	9.0	9.9	0.91 (0.60, 1.37)	0.82 (0.52, 1.29)	0.82 (0.44, 1.51)
Total effect	310	81,082	8.4	9.6	0.88 (0.59, 1.31)	0.83 (0.51, 1.35)	0.83 (0.43, 1.60)
**Vector control intervention** (RAVC vs. no RAVC)							
Direct effect	310	8,252	8.9	7.6	1.17 (0.62, 2.23)	1.35 (0.54, 3.34)	1.35 (0.43, 4.25)
Spillover effect	310	72,830	12.9	18.5	0.69 (0.47, 1.03)	0.68 (0.46, 1.00)	0.68 (0.36, 1.30)
Total effect	310	81,082	12.5	17.4	0.72 (0.49, 1.06)	0.75 (0.48, 1.20)	0.75 (0.33, 1.70)
**Combined intervention** (rfMDA + RAVC vs. RACD only)							
Direct effect	143	3,914	6.4	7.4	0.87 (0.32, 2.41)	1.03 (0.22, 4.81)	1.03 (0.19, 5.58)
Spillover effect	143	38,048	11.2	18.1	0.62 (0.34, 1.13)	0.57 (0.41, 0.80)	0.57 (0.42, 0.79)
Total effect	143	41,962	10.8	17.1	0.63 (0.35, 1.12)	0.37 (0.22, 0.63)	0.37 (0.18, 0.79)

Supplementary Table 4. Direct effect, spillover effect, and total effect estimates on cumulative incidence of malaria infection
For rfMDA and RACD arms, the analysis includes the period from 0-35 days following index case detection for direct effects and 21-56 days for spillover effects. For rfMDA+RAVC and RAVC only arms, the analysis includes the period from 0-6 months following index case detection for direct effects and 17 days to 6 months for spillover effects. Total effects analyses include the person-time for the direct effects and spillover effects analyses. Direct effect includes intervention recipients in the target zone. Spillover effect analyses includes intervention non-recipients up to 1km from an index case. Total effect includes all individuals (intervention recipients and non-recipients) up to 1km from index case. Models were fit with hierarchical targeted maximum likelihood. All outcome models were fit with cohort-level data except for models of spillover effects of rfMDA + RAVC vs. RACD only. Covariates were screened separately for each model using a likelihood ratio test. We separately fit individual- and cohort-level outcome models and report the model with the smaller cross-validated mean squared error. All models except spillover effects of the chemoprevention and combined interventions were fit on cohort-level data.

	Below median		Above median	
	**Minimum**	**Maximum**	**Minimum**	**Maximum**
Malaria incidence per 1,000 in 2016	0.0	13.9	14.9	293.3
Pre-season indoor residual spray coverage 2016 (%)	27.2	77.3	77.9	100
Median daytime land surface temperature (C)	28.6	31.1	31.1	33.4
Median monthly rainfall November 2016-April 2017 (mm)	18.4	23.7	23.7	26.7
Median enhanced vegetative index January 2017-July 2017	0.09	0.15	0.15	0.31
Median elevation (m)	387	541	544	1124
Cohort-level treatment coverage (%)	0.0	8.3	8.3	97.4

Supplementary Table 5. Range above and below median value in each enumeration area for subgroup variables

		N individuals			N cohorts		
**Sensitivity analysis**	**Intervention**	**Direct effect**	**Spillover effect**	**Total effect**	**Direct effect**	**Spillover effect**	**Total effect**
Primary analysis	Chemoprevention	8252	72830	81082	310	310	310
	Vector control	8252	72830	81082	310	310	310
	Combined	3914	38048	41962	143	143	143
Alternative observation	Chemoprevention	8252	72830	81082	310	310	310
	Vector control	8252	72830	81082	310	310	310
	Combined	3914	38048	41962	143	143	143
No overlap spillover	Chemoprevention	4728	42141	46869	182	182	182
	Vector control	6080	56159	62239	235	235	235
	Combined	2917	27973	30890	107	107	107
No overlap target	Chemoprevention	5875	57600	63475	222	222	222
	Vector control	7898	72056	79954	292	292	292
	Combined	3769	37725	41494	135	135	135

Supplementary Table 6. Number of individuals and cohorts included in sensitivity analyses

	Primary analysis		Sensitivity analysis with shorter observation period	
	Target areas	Spillover zone	Target areas	Spillover zone
**Chemoprevention intervention** (rfMDA vs. RACD)	32.0	28.9	21.0	17.4
**Vector control intervention** (RAVC vs. no RAVC)	59.2	47.5	52.5	41.2
**Combined intervention** (rfMDA + RAVC vs. RACD only)	60.5	28.1	57.8	24.5

Supplementary Table 7. Percentage of cohorts overlapping with other cohorts
Overlap in target area was defined as index cases that triggered interventions located within <1km of each other and observation periods that temporally overlapped with another cohort's. Overlap in spillover zones was defined as index cases that triggered interventions located within 1-2km of each other and observation periods that temporally overlapped with another cohort's. The denominator was the total cohorts included in each analysis.

	N		Prevalence		Prevalence ratio (95% CI)	
	Intervention arm	Reference arm	Intervention arm	Reference arm	Unadjusted	Adjusted
**Chemoprevention intervention** (rfMDA vs. RACD)						
Direct effect	1537	1835	0.029	0.033	0.90 (0.61, 1.31)	0.84 (0.53, 1.32)
Spillover effect	244	229	0.025	0.087	0.28 (0.12, 0.69)	--
Total effect	1781	2064	0.029	0.039	0.74 (0.52, 1.04)	0.79 (0.51, 1.19)
**Vector control intervention** (RAVC vs. no RAVC)						
Direct effect	1710	1662	0.026	0.037	0.70 (0.48, 1.03)	0.78 (0.51, 1.21)
Spillover effect	195	278	0.051	0.058	0.89 (0.41, 1.92)	--
Total effect	1905	1940	0.028	0.040	0.71 (0.51, 1.01)	0.64 (0.43, 0.96)
**Combined intervention** (rfMDA + RAVC vs. RACD only)						
Direct effect	758	883	0.017	0.033	0.52 (0.27, 1.00)	--
Spillover effect	118	152	0.017	0.079	0.21 (0.05, 0.94)	--
Total effect	876	1035	0.017	0.040	0.43 (0.24, 0.78)	--

Supplementary Table 8. Direct effect, spillover effect, and total effect estimates on malaria prevalence measured by qPCR
Prevalence was measured in a cross-sectional survey in a random sample of households at the end of the malaria season. Analyses were restricted to individuals located within 3 km of at least one intervention recipient. Direct effects include individuals with any intervention recipients within 500m, spillover effects include individuals with no intervention recipients < 500m and any intervention recipients 500m-3km, and total effects include individuals with any intervention recipients <3km during the study. Prevalence ratios were estimated using TMLE with individual-level data, and standard errors were adjusted for clustering at the enumeration area level. Adjusted models were not fit if there were fewer than 30 observations within strata of the intervention and outcome.

	N households		Prevalence		Unadjusted Prevalence Ratio (95% CI)
	Intervention arm	Reference arm	Intervention arm	Reference arm	
**Chemoprevention intervention** (rfMDA vs. RACD)					
Direct effect	456	506	0.018	0.018	0.99 (0.38, 2.54)
Spillover effect	72	69	0.000	0.043	0.00 (0.00, 0.00)
Total effect	528	575	0.015	0.021	0.73 (0.30, 1.76)
**Vector control intervention** (RAVC vs. no RAVC)					
Direct effect	481	481	0.012	0.023	0.55 (0.20, 1.46)
Spillover effect	65	76	0.015	0.026	0.58 (0.05, 6.35)
Total effect	546	557	0.013	0.023	0.55 (0.22, 1.37)
**Combined intervention** (rfMDA + RAVC vs. RACD only)					
Direct effect	219	244	0.005	0.016	0.28 (0.03, 2.48)
Spillover effect	36	40	0.000	0.050	0.00 (0.00, 0.00)
Total effect	255	284	0.004	0.021	0.19 (0.02, 1.53)

Supplementary Table 9. Direct effect, spillover effect, and total effect estimates on household-level malaria prevalence of measured by qPCR

Prevalence was measured in a cross-sectional survey in a random sample of households at the end of the malaria season. Analyses were run at the household level. Household-level malaria prevalence was the percentage of households with more than one malaria case detected in the prevalence survey by qPCR. Direct effects include households with any intervention recipients within 500m, spillover effects include households with no intervention recipients < 500m and any intervention recipients 500m-3km, and total effects include households with any intervention recipients <3km during the study. Prevalence ratios were estimated using TMLE with household-level data. Adjusted models were not fit because there were fewer than 30 observations within strata of the intervention and outcome.

	N		Prevalence		Prevalence ratio (95% CI)	
	Intervention arm	Reference arm	Intervention arm	Reference arm	Unadjusted	Adjusted
**Chemoprevention intervention** (rfMDA vs. RACD)						
Direct effect	1316	1611	0.215	0.285	0.75 (0.66, 0.86)	0.84 (0.71, 1.00)
Spillover effect	198	182	0.227	0.225	1.01 (0.69, 1.46)	1.32 (0.73, 2.41)
Total effect	1514	1793	0.217	0.279	0.78 (0.69, 0.88)	0.85 (0.73, 0.99)
**Vector control intervention** (RAVC vs. no RAVC)						
Direct effect	1475	1452	0.241	0.267	0.90 (0.80, 1.02)	0.90 (0.79, 1.04)
Spillover effect	133	247	0.188	0.247	0.76 (0.50, 1.15)	--
Total effect	1608	1699	0.236	0.264	0.90 (0.80, 1.01)	0.88 (0.76, 1.01)
**Combined intervention** (rfMDA + RAVC vs. RACD only)						
Direct effect	634	770	0.194	0.295	0.66 (0.54, 0.80)	--
Spillover effect	81	130	0.136	0.208	0.66 (0.55, 0.80)	--
Total effect	715	900	0.187	0.282	0.65 (0.34, 1.25)	--

Supplementary Table 10. Direct effect, spillover effect, and total effect estimates on Etramp5.Ag1 seroprevalence
Prevalence was measured in a cross-sectional survey in a random sample of households at the end of the malaria season. Analyses were restricted to individuals located within 3 km of at least one intervention recipient. Direct effects include individuals with any intervention recipients within 500m, spillover effects include individuals with no intervention recipients < 500m and any intervention recipients 500m-3km, and total effects include individuals with any intervention recipients <3km during the study. Prevalence ratios were estimated using TMLE with individual-level data, and standard errors were adjusted for clustering at the enumeration area level. Adjusted models were not fit if there were fewer than 30 observations within strata of the intervention and outcome.

	Intervention cost	N individuals	Prevalence	Prevalent cases	Total prevalent cases averted (95% CI)	ICER (95% CI)	% change in ICER
RACD	$354,750	9,394	0.039	364	(ref)	(ref)	
rfMDA	$368,321	9,417	0.029	270	94 (89, 100)	$144 ($136, $153)	-11%
No RAVC	$261,409	9,400	0.040	373	(ref)	(ref)	
RAVC	$461,661	9,407	0.028	267	106 (95, 118)	$1,882 ($1,679, $2,111)	-30%
RACD only	$127,312	4,516	0.040	179	(ref)	(ref)	
rfMDA+RAVC	$234,223	4,500	0.017	77	102 (87, 117)	$1,050 ($915, $1,231)	-42%

Supplementary Table 11. Cost-effectiveness analysis

Incremental cost-effectiveness ratios (ICERs) were estimated using prevalent cases averted from hierarchical TMLE models for prevalence measured by qPCR. The number of prevalent cases averted equaled the produce of the difference in prevalence between arms among intervention recipients and non-recipients by the estimated population size within target areas vs. spillover zones. The incremental cost effectiveness ratio is the ratio of the difference in cost between arms by the difference in prevalent cases averted in both target area and spillover zones within 3 km of index cases for rfMDA + RAVC vs. RACD. The % change in ICER compares the ICER accounting for spillover effects to the original trial's ICER estimate reported in Ntuku et al., 2022 10.1136/bmjopen-2021-049050.
