# Supplementary material for: Extension of efficacy range for targeted malaria-elimination interventions due to spillover effects
Source: Nat Med. 2024 Jul 4;30(10):2813–20. doi: 10.1038/s41591-024-03134-z (PMC11483210; doi:10.1038/s41591-024-03134-z)
Supplement: Supplementary file 2 — Reporting Summary [file 41591_2024_3134_MOESM2_ESM.pdf]

Corresponding author(s): Jade Benjamin-Chung
Last updated by author(s): Jun 10, 2024

Reporting Summary

Nature Portfolio wishes to improve the reproducibility of the work that we publish. This form provides structure for consistency and transparency in reporting. For further information on Nature Portfolio policies, see our Editorial Policies and the Editorial Policy Checklist.

Statistics

For all statistical analyses, confirm that the following items are present in the figure legend, table legend, main text, or Methods section.

n/a	Confirmed	
☐	☒	The exact sample size (*n*) for each experimental group/condition, given as a discrete number and unit of measurement
☒	☐	A statement on whether measurements were taken from distinct samples or whether the same sample was measured repeatedly
☐	☒	The statistical test(s) used AND whether they are one- or two-sided<br>*Only common tests should be described solely by name; describe more complex techniques in the Methods section.*
☐	☒	A description of all covariates tested
☐	☒	A description of any assumptions or corrections, such as tests of normality and adjustment for multiple comparisons
☐	☒	A full description of the statistical parameters including central tendency (e.g. means) or other basic estimates (e.g. regression coefficient) AND variation (e.g. standard deviation) or associated estimates of uncertainty (e.g. confidence intervals)
☐	☒	For null hypothesis testing, the test statistic (e.g. *F*, *t*, *r*) with confidence intervals, effect sizes, degrees of freedom and *P* value noted<br>*Give P values as exact values whenever suitable.*
☒	☐	For Bayesian analysis, information on the choice of priors and Markov chain Monte Carlo settings
☐	☒	For hierarchical and complex designs, identification of the appropriate level for tests and full reporting of outcomes
☐	☒	Estimates of effect sizes (e.g. Cohen's *d*, Pearson's *r*), indicating how they were calculated

Our web collection on statistics for biologists contains articles on many of the points above.

Software and code

Policy information about availability of computer code

Data collection	Data in the original trial was collected with Open Data Kit.
Data analysis	Replication scripts are available at https://zenodo.org/doi/10.5281/zenodo.11410094. Software packages for statistical analyses include R version 4.1.0 and the following R packages: SuperLearner version 2.0-28.1; sl3 version 1.4.4; glmnet version 4.1-2; xgboost version 1.4.1.1; tmle3 version 0.2.0.

For manuscripts utilizing custom algorithms or software that are central to the research but not yet described in published literature, software must be made available to editors and reviewers. We strongly encourage code deposition in a community repository (e.g. GitHub). See the Nature Portfolio guidelines for submitting code & software for further information.

Data

Policy information about availability of data

All manuscripts must include a data availability statement. This statement should provide the following information, where applicable:

- Accession codes, unique identifiers, or web links for publicly available datasets
- A description of any restrictions on data availability
- For clinical datasets or third party data, please ensure that the statement adheres to our policy

This study is a secondary analysis of data from a cluster-randomized trial of focal malaria interventions conducted in Zambezi region of Namibia (NCT02610400). Data from the original trial is available at https://clinepidb.org/ce/app/workspace/analyses/DS_f559aee789. Data can also be requested from Michelle Hsiang

(michelle.hsiang@ucsf.edu). All data requests that comply with limitations contained in the informed consent form signed by the participants of the trial will be granted access.

Research involving human participants, their data, or biological material

Policy information about studies with human participants or human data. See also policy information about sex, gender (identity/presentation), and sexual orientation and race, ethnicity and racism.

Reporting on sex and gender	We assessed effect modification by gender for spillover effects. The sample size was too small to do so for direct effects.
Reporting on race, ethnicity, or other socially relevant groupings	Analyses did not account for race/ethnicity. The study population is from Namibia and is of African ancestry.
Population characteristics	The study population is individuals living in Zambezi region of Namibia, where a cluster-randomized controlled trial was conducted. All individuals in the study clusters were eligible for inclusion in this analysis. The study population included individuals of all ages and genders. The population included a mix of individuals with current diagnosed malaria infection and unknown malaria infection status at the start of the study.
Recruitment	The trial defined clusters based on census enumeration areas that were within the catchment area of study health care facilities. Enumeration areas were eligible for inclusion in the trial if they 1) were located in the catchment areas of 11 health facilities, 2) had complete incidence data from 2012-13, and 3) had at least one incident case during the trial.
Ethics oversight	The trial protocol was approved by the Namibia Ministry of Health and Social Services (17/3/3) and the Institutional Review Boards at the University of California San Francisco (15–17422) and London School of Hygiene & Tropical Medicine (10411). The secondary analysis protocol was approved by the Stanford University Institutional Review Board (60708).

Note that full information on the approval of the study protocol must also be provided in the manuscript.

Field-specific reporting

Please select the one below that is the best fit for your research. If you are not sure, read the appropriate sections before making your selection.

☐ Life sciences ☒ Behavioural & social sciences ☐ Ecological, evolutionary & environmental sciences

For a reference copy of the document with all sections, see nature.com/documents/nr-reporting-summary-flat.pdf

Behavioural & social sciences study design

All studies must disclose on these points even when the disclosure is negative.

Study description	This study was a secondary analysis of quantitative data from a cluster-randomized trial of focal malaria interventions conducted in Zambezi region of Namibia from January 1 to December 31, 2017.
Research sample	The analysis used data from a cluster-randomized trial of focal malaria interventions conducted in Zambezi region of Namibia from January 1 to December 31, 2017. The study population included all individuals in study clusters. These individuals are representative of low malaria transmission populations in Namibia. This sample was chosen because reactive, focal interventions are most appropriate for low transmission settings approach malaria elimination. All individuals in the study clusters were eligible for inclusion in this analysis. The study population included individuals of all ages and genders. The population included a mix of individuals with current diagnosed malaria infection and unknown malaria infection status at the start of the study.
Sampling strategy	We included all individuals recorded in the baseline geographic census, subsequent malaria cases detected through surveillance, and individuals sampled in the cross-sectional survey near the end of the trial. We did not restrict to any subsample of participants from the original trial, and all individuals resided within the original trial's study clusters.
Data collection	No new data was collected for this study. The manuscript Methods section includes a brief summary of data collection in the original trial. Briefly, the original trial collected a geographical census at baseline; collected demographic, epidemiologic, and clinical data on malaria index cases and intervention recipients during the trial; and collected a cross-sectional survey at endline. It was not practical to blind study participants or field staff to intervention assignment, but laboratory analyses and primary statistical analyses were blinded.
Timing	January 1 to December 31, 2017
Data exclusions	We excluded 32 cohorts from the incidence analysis for which it was not possible to merge intervention recipient geocoordinates with index data geocoordinates. We did not exclude any data from the prevalence analysis.
Non-participation	This is an analysis of an secondary dataset, so there were no new participants in this study.
Randomization	The original trial used a two-by-two factorial design. The trial allocated 56 study clusters to study arms using restricted randomization. Arms included receive reactive case detection (RACD) only, reactive focal mass drug administration (rfMDA) only,

RACD + reactive vector control (RAVC), and rfMDA + RAVC. Restriction criteria included mean annual incidence in 2013 and 2014, population size, population density, and mean distance from the household to a health-care facility.

Reporting for specific materials, systems and methods

We require information from authors about some types of materials, experimental systems and methods used in many studies. Here, indicate whether each material, system or method listed is relevant to your study. If you are not sure if a list item applies to your research, read the appropriate section before selecting a response.

Materials & experimental systems

n/a	Involved in the study
☒	☐ Antibodies
☒	☐ Eukaryotic cell lines
☒	☐ Palaeontology and archaeology
☒	☐ Animals and other organisms
☐	☒ Clinical data
☒	☐ Dual use research of concern
☒	☐ Plants

Methods

n/a	Involved in the study
☒	☐ ChIP-seq
☒	☐ Flow cytometry
☒	☐ MRI-based neuroimaging

Clinical data

Policy information about clinical studies

All manuscripts should comply with the ICMJE guidelines for publication of clinical research and a completed CONSORT checklist must be included with all submissions.

Clinical trial registration: NCT02610400

Study protocol: http://dx.doi.org/10.1136/bmjopen-2017-019294

Data collection: This study was a secondary analysis of a cluster-randomized trial of focal malaria interventions conducted in Zambezi region of Namibia from January 1 to December 31, 2017. The region has seasonal malaria transmission that peaks between January and June. Plasmodium falciparum is the dominant species, and annual Pf incidence was less than 15 per 1,000 from 2010-2015. In 2016, the incidence was 32.5 per 1,000 following an outbreak. In 2015, prevalence measured by loop-mediated isothermal amplification was 2.2%. In the study site, the Namibia Ministry of Health and Social Services routinely delivered case management and annual preseason household IRS with dichlorodiphenyltrichloroethane, with the exception of a small number of structures that were sprayed with deltamethrin. In addition, they offered reactive case detection within 500 m of confirmed malaria cases, which included testing with rapid diagnostic tests and treatment with artemether-lumefrantrine and single-dose primaquine for those who tested positive.

Outcomes: The primary outcome was incidence of malaria infection. Secondary outcomes were prevalence of current infection and seroprevalence. During the trial, trial staff extracted data on confirmed incident malaria cases and travel history from the rapid reporting system. At the end of malaria season between May and August 2017, the study team collected an endline cross-sectional survey to measure infection prevalence. Field staff collected dried blood spots on filter paper (Whatman 3 Corporation, Florham Park, NJ, USA) by finger prick from consenting individuals, and qPCR was performed targeting the acidic terminal sequence of the var gene. Field staff also collected 250 ml of whole blood in BD Microtainer tubes with EDTA additive (Becton, Dickinson and Corporation, Franklin Lakes, NJ, USA) for serological analyses. Using human plasma, Luminex assays were performed to detect malaria antigens using previously described procedures.
